# Ninth International Medical Congress

**Published:** 1885-04

**Authors:** 


NINTH INTERNATIONAL MEDICAL CONGRESS. WASHINGTON, D. C., 1887.—*Section on Dental and Oral Surgery.*—Below appears the list of the officers of the Section on Dental and Oral Surgery of the Ninth International Medical Congress to meet in Washington, D. C. in 1877, and which has just been officially announced. The selection of such a noble body of men to represent the dentists of America reflects credit upon the judgment and foresight of the Executive committee of the Congress, and their efforts to elect gentlemen that would be representative of, and a credit to our specialty, should receive, and no doubt will, the hearty support of every dentist in the land.

The gentlemen chosen are "good men and true," and the honor of American dentistry can be safely entrusted to their keeping.

PRESIDENT.

Jonathan Taft, M. D., - - - - - Cincinnati.

VICE-PRESIDENTS.

W. W. Allport, M. D., - - - - - - Chicago.
William H. Dwinelle, M. D., - - - - New York.
Jacob L. Williams, M. D., - - - - - Boston.

SECRETARIES.

Edward A. Bogue, M. D., - - - - - New York.
George H. Cushing, M. D., - - - - - Chicago.

COUNCIL.

W. C. Barrett, M. D., - - - - - - - Buffalo.
Thomas Fillebrown, M. D., - - - - - Boston.
F. J. S. Gorgas, M. D., - - - - - Baltimore.
Edward Maynard, M. D., - - - - Washington.
H. J. McKellops, D. D. S., - - - - - St. Louis
W. H. Morgan, M. D., - - - - - - Nashville.
C. Newlin Peirce, D. D. S. - - - Philadelphia.
L. D. Shepard, D. D. S., - - - - . - Boston.
James Truman, D. D. S., - - - - Philadelphia.
J. W. White, M. D., - - - - - Philadelphia.

—*The Archives of Dentistry.*

